# Origin of Heat Capacity Changes in a “Nonclassical” Hydrophobic Interaction

**DOI:** 10.1002/cbic.200700281

**Published:** 2007-07-12

**Authors:** Neil R Syme, Caitriona Dennis, Simon E V Phillips, Steve W Homans

**Keywords:** entropy, heat capacity, hydrophobic effects, solvation, thermodynamics

The early work of Edsall[1] illustrated the effect of nonpolar groups in raising the apparent heat capacities of solutes in aqueous solution. Subsequently, the hydrophobic effect has been well established as the result of the formation of structured water around such groups.[2–5] While the nature of this structuring remains a topic of debate,[6] it is universally accepted that these water molecules possess a higher heat capacity and a lower entropy than bulk water. Consequently, hydrophobic interactions, in which nonpolar surfaces are shielded from bulk water, are classically characterized by a favourable entropic binding signature together with a negative change in heat capacity at constant pressure (Δ*C*_p_).[3–5, 7] Nonetheless, the foundations of the latter in solvent reorganisation are not universally accepted.[8]

Recently, we observed a paradoxical enthalpy-driven thermodynamic binding signature[9] in studies on a model ligand–protein interaction, namely the association of small hydrophobic ligands within the hydrophobic binding pocket of recombinant mouse major urinary protein (rMUP).[10] This binding signature has been observed in a number of hydrophobic molecular interactions (reviewed by Meyer et al.[11]) and has led to the concept of the “nonclassical” hydrophobic interaction. However, the thermodynamic relationship between the “nonclassical” and “classical” hydrophobic interaction has remained obscure, especially since the former typically exhibits the negative change in heat capacity of the latter; this suggests that the molecular basis of each lies in solvent reorganization. In the case of rMUP we found that the binding pocket is suboptimally hydrated,[12] a phenomenon that is increasingly being reported in other proteins.[13] Under these circumstances, there exists an imbalance between solute–solute dispersion interactions following the association, versus solute–solvent dispersion interactions that exist prior to the association. Moreover, the favourable entropic contribution that results from the expulsion of solvent water molecules is small, and leads to a thermodynamic binding signature that is enthalpy driven.[12, 14] However, a negative change in heat capacity on binding ($\Delta C_{\text{p}}^{\text{b}}$) is nonetheless observed in rMUP–ligand interactions, and is of similar or greater magnitude to that observed in typical ligand–protein interactions (Table 1).[15–17] The serendipitous discovery that rMUP binds members of the primary aliphatic alcohol series as surrogate ligands[18] permits a rigorous quantitative assessment of the solvation contribution to Δ*C*_p_, since the hydration thermodynamics of these ligands are very well characterized.[19, 20]

**TABLE 1:** $\Delta C_{\text{p}}^{\text{b}}$ values for a number of typical protein–ligand interactions.

System	$\Delta C_{\text{p}}^{\text{b}}$ [J K^−1^ mol^−1^]
rMUP–heptan-1-ol (this study)	−663±51
carbonic anhydrase–*p*-gly3-benzenesulfonamide[17]	−75±40
arabinose binding protein–d-galactose[16]	−656±57
trypsin–*p*-*N*-hexyl-benzamidinium chloride[15]	−849±10

We measured the temperature dependence of the standard enthalpy of binding of hexan-1-ol, heptan-1-ol and octan-1-ol to rMUP using conventional isothermal titration calorimetry (ITC) experiments.[21] A linear fit of these data (Figure 1) resulted in $\Delta C_{\text{p}}^{\text{b}}$ values, shown in Table 2, which are compared with literature data for the desolvation heat capacities of the respective ligands ($\Delta C_{\text{p}}^{\text{desolv}}$).[19, 20] It can be seen that about 80 % of the change in heat capacity on binding can be accounted for by the ligand desolvation process, and this change is the same for each complex within error. Moreover, the excess change in heat capacity ($\Delta C_{\text{p}}^{\text{b}}$−$\Delta C_{\text{p}}^{\text{desolv}}$) is the same for each complex within error, and amounts to about −100 J mol^−1^ K^−1^.

**Figure 1:**
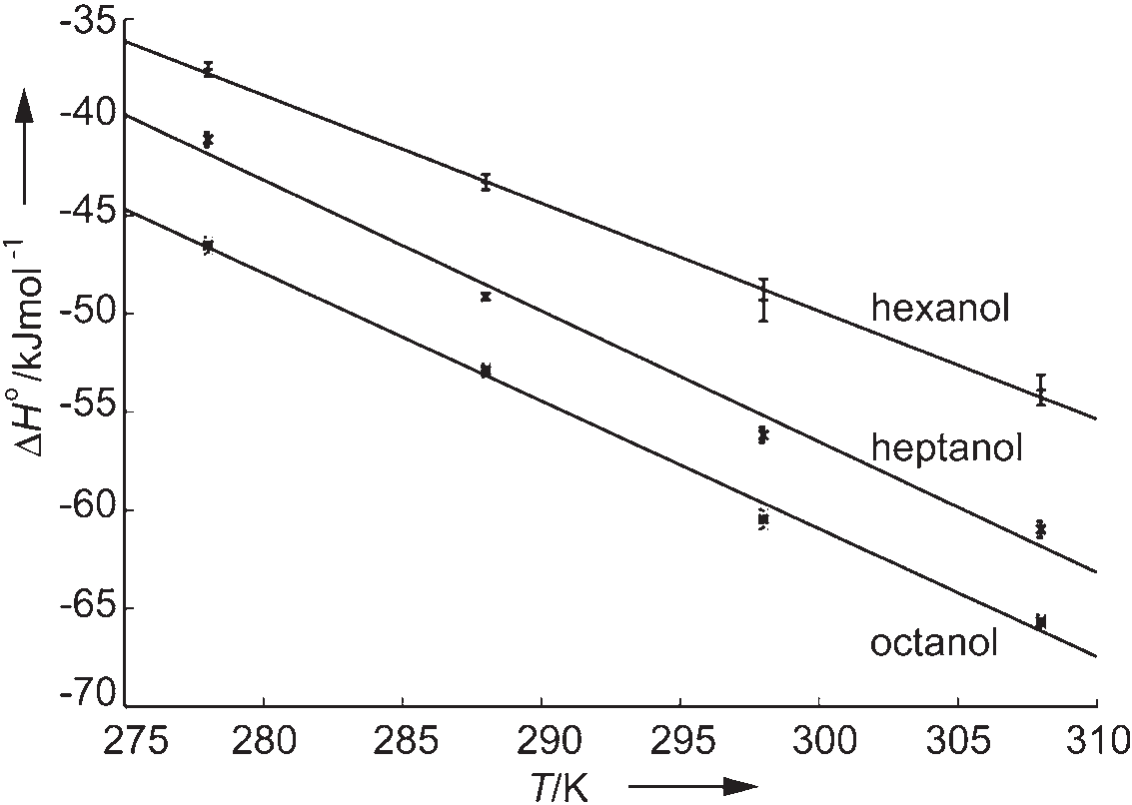
Temperature dependence of $\Delta H_{\text{b}}^{\text{o}}$ for binding of hexanol, heptanol and octanol to rMUP.

**TABLE 2:** Binding and desolvation heat capacities [J K^−1^ mol^−1^] for primary alcohols in association with rMUP.

Ligand	$\Delta C_{\text{p}}^{\text{b}}$[a]	$\Delta C_{\text{p}}^{\text{desolv}}$[b]	$\Delta C_{\text{p}}^{\text{b}}$−$\Delta C_{\text{p}}^{\text{desolv}}$	$\Delta C_{\text{p}}^{\text{desolv}}$/$\Delta C_{\text{p}}^{\text{b}}$
hexanol	−553±15	−460±20	−93±25	0.83±0.4
heptanol	−663±56	−520±30	−143±64	0.78±0.08
octanol	−646±27	−570±30	−76±40	0.88±0.06

[a] Change in heat capacity upon binding of indicated ligand to rMUP. Reported errors derive from errors in the linear fit of the temperature dependence of the standard enthalpy of binding as a function of temperature.

[b] Standard-state desolvation heat capacity of the respective ligand with associated errors in parentheses, which was determined from literature values for solvation heat capacities. Values reported are mean values from several sources, as described.[20]

We sought to determine whether this excess could be attributed to expulsion of residual ordered solvent water molecules from the protein binding pocket. Our original crystal structure of rMUP in the absence of ligand[10] (PDB ID: 1QY0) was not suitable for the observation of ordered solvent water molecules within the binding pocket due to the presence of cryoprotectant glycerol. We, therefore, solved the structure in the presence of an alternative cryoprotectant, paratone-N, which does not bind in the binding pocket of the protein. The structure revealed the presence of four ordered solvent water molecules in the apo structure (Figure 2).

**Figure 2:**
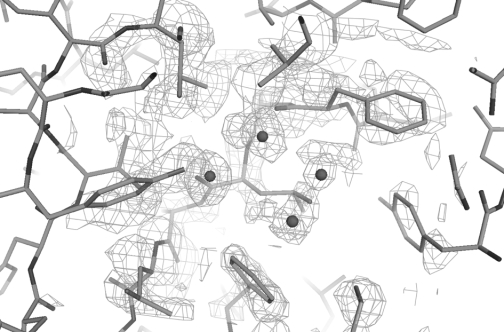
Electron density in the binding pocket of apo rMUP, which shows four ordered water molecules as spheres.

Privalov and Gill have studied the hydration heat capacities of a number of nonpolar gases and liquid hydrocarbons.[22] By assuming that a given water molecule in contact with each solute occupies an area of about 9 Å^2^, they concluded that the increase in heat capacity on solvation of hydrophobic groups amounts to about 13 J mol^−1^ K^−1^ per water molecule.[22] These data suggest that the contribution to $\Delta C_{\text{p}}^{\text{b}}$ from loss of ordered water molecules from the protein-binding pocket is at most about −50 J mol^−1^ K^−1^, since the crystal structures of the rMUP–alcohol complexes also contain between one and three ordered water molecules.[18] There exists the formal possibility that disordered water molecules within the binding pocket might also contribute to the thermodynamic binding signature. However, previous molecular dynamics simulations on uncomplexed rMUP suggested that on average only 3.5 water molecules are present in the binding pocket,[12] which is in good agreement with the X-ray data.

We conclude that the characteristic negative change in heat capacity observed in rMUP–ligand complexation is largely determined by ligand desolvation, with minor contribution from desolvation of the protein. In principle a contribution to the excess change in heat capacity $\Delta C_{\text{p}}^{\text{b}}$−$\Delta C_{\text{p}}^{\text{desolv}}$ might arise either from a change in vibrational degrees of freedom of the protein or ligand, or both. This contribution is expected to be small[5] but is currently under investigation. We further conclude that despite the very different thermodynamic signatures of the “classical” and “nonclassical” hydrophobic effect, the molecular basis of each lies in solvent reorganization. In the case of rMUP, the entropic contribution that would normally be expected to arise from protein desolvation is not present. Instead, the favourable enthalpic contribution that arises from solute–solute dispersion interactions, which also indirectly arises from solvation (or desolvation in this case), becomes the dominant term.

## Experimental Section

**ITC experiments**: Recombinant MUP was over-expressed and purified as described previously.[10] ITC experiments were carried out by using a Microcal VP-ITC unit. Before use, all protein was precipitated in ethanol to remove any endogenous ligands, then redissolved, dialysed against PBS (pH 7.4) and degassed under reduced pressure. Protein concentrations were measured prior to experiments by using UV absorption (MUP *ɛ*_280_=10 650 m^−1^ cm^−1^). Protein (50 μm) was titrated with hexanol (750 μm) or heptanol (750 μm), or alternatively protein (25 μm) was titrated with octanol (250 μm). A lower concentration of octanol was employed due to its reduced solubility. Titration consisted of 30 injections (1×2 μL followed by 29×5 μL) at 4 min intervals. The initial injection (2 μL) was discarded during fitting to allow for equilibration of ligand–receptor at the needle tip. Experiments were carried out at 278, 288, 298 and 308 K for each of the alcohols hexanol, heptanol and octanol. Data were fitted by using the One-Site model present in Origin 5.0 (Microcal Inc.), which is based on the Wiseman isotherm.[21, 23]

**X-ray crystallography**: Crystals of recombinant MUP were grown as described previously.[24] A single crystal was immersed in Paratone-N (Hampton Research, USA) prior to being frozen in liquid nitrogen. Data were recorded at 100 K with an R-AXIS IV^++^ image plate detector mounted on a Rigaku RU-H3R rotating anode X-ray generator, and integrated and reduced by using MOSFLM[25] and SCALA (CCP4, Collaborative Computational Project, Number 4, 1994), respectively. The structure of MUP-1 (PDB ID: 1QY0) was rigid-body refined into the unit cell (*a*=*b*=53.79 Å, *c*=137.52 Å, space group, *P*4_3_2_1_2) and refined by using REFMAC.[25, 26] Further model building and water placement was carried out in COOT.[27] Crystal coordinates have been deposited in the RCSB protein databank, ID: 2OZQ.
