# Supplementary material for: Origin of Heat Capacity Changes in a “Nonclassical” Hydrophobic Interaction
Source: Chembiochem. 2007 Jul 12;8(13):1509–11. doi: 10.1002/cbic.200700281 (PMC2435424; doi:10.1002/cbic.200700281)
Supplement: Supplementary file 1 [file cbic0008-1509-SD1.pdf]

CHEMBIOCHEM

Supporting Information

© Copyright Wiley-VCH Verlag GmbH & Co. KGaA, 69451 Weinheim, 2007

© Copyright Wiley-VCH Verlag GmbH & Co. KGaA, 69451 Weinheim, 2007

CHEMBIOCHEM

Supporting Information

for

Origin of Heat Capacity Changes in a "Nonclassical" Hydrophobic Interaction

Neil R. Syme, Caitriona Dennis, Simon E. V. Phillips, Steve W. Homans*

Table S1. Data Collection and Refinement statistics. Values in parentheses are for the highest resolution shell [1.9 – 1.8 Å].

		Apo-rMUP
Wavelength [Å]		1.54
Resolution range [Å]		42.4–1.8
Unique reflections		16 532 (2623)
Completeness (%)		86 (95.8)
Multipicity		5.2 (5.5)
R_{sym}[a]		11 (37.8)
Unit cell dimensions [Å], [°]		*a* = *b* = 53.79, *c* = 137.52; *a* = *ß* = *?* = 90
Space group		$P4_32_12$
R_{work} / R_{free}		18 / 23
rmsd from ideal:	bond length [Å]	0.012
	bond angles [°]	1.205

[a] $R_{sym} = S_{hkl}S_i(I_i(hkl) – I_{mean}(hkl))/S_{hkl}S_i(I_i(hkl))$.
